# Supplementary figures and images for: Phenotype and molecular signature of CD8+ T cell subsets in T cell- mediated rejections after kidney transplantation
Source: PLoS One. 2020 Jun 12;15(6):e0234323. doi: 10.1371/journal.pone.0234323 (PMC7292394; doi:10.1371/journal.pone.0234323)

Supplement Figure 1

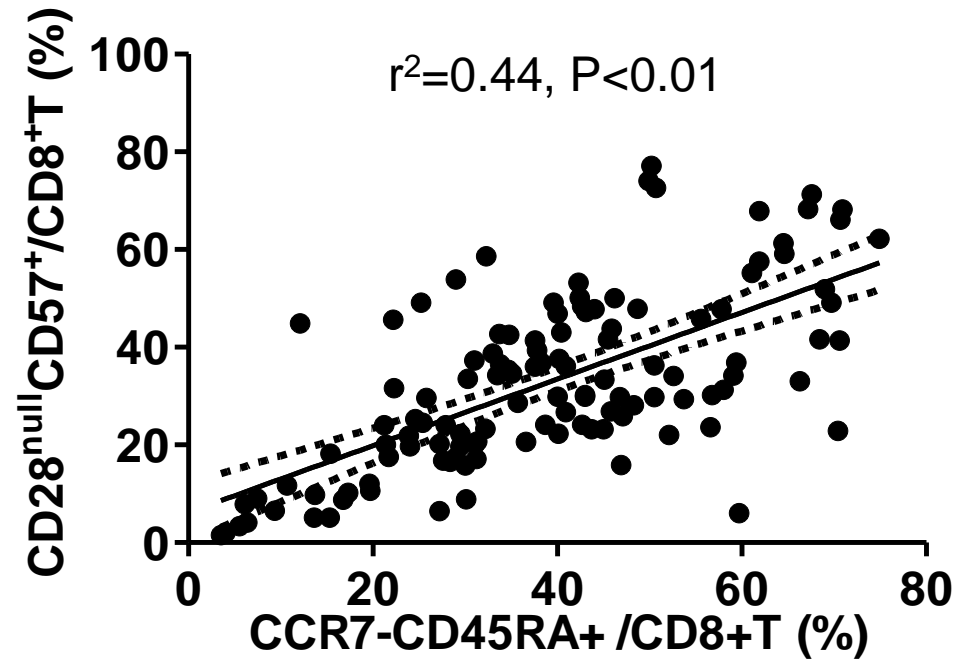

Supplement: S1 Fig — The proportion (%) of CCR7-CD45RA+CD8+ T cells showed a significant correlation with the proportion (%) of CD28nullCD57+CD8+ T cells (p < 0.001, r2 = 0.44). (PDF) [file pone.0234323.s001.pdf]
