# Supplementary material for: Phenotype and molecular signature of CD8+ T cell subsets in T cell- mediated rejections after kidney transplantation
Source: PLoS One. 2020 Jun 12;15(6):e0234323. doi: 10.1371/journal.pone.0234323 (PMC7292394; doi:10.1371/journal.pone.0234323)
Supplement: S1 Table — (PDF) [file pone.0234323.s002.pdf]

Supplementary Table 1. Significantly changed genes along *ex vivo* CCR7^{+}CD8^{+}T cells

Gene symbol	Score(d)	q-value (%)
CA6	3.957	0
MGC15705	3.708	0
EDAR	3.674	0
RAB3IP	3.625	0
C2orf40	3.605	0
NOG	3.585	0
GCNT4	3.442	0
ACACB	3.401	0
LEF1	3.38	0
TFDP3	3.335	4.136
C2orf89	3.264	4.136
ACVR1C	3.161	4.136
LRRN3	3.147	4.136

+13 / -0
