# Supplementary material for: Phenotype and molecular signature of CD8+ T cell subsets in T cell- mediated rejections after kidney transplantation
Source: PLoS One. 2020 Jun 12;15(6):e0234323. doi: 10.1371/journal.pone.0234323 (PMC7292394; doi:10.1371/journal.pone.0234323)
Supplement: S2 Table — (PDF) [file pone.0234323.s003.pdf]

**Supplementary Table 2. Significantly changed genes along *ex vivo* CCR7$^-$CD45RA$^+$CD8$^+$T cells**

| Gene Symbol | Score (d) | q-value (%) |
|---|---|---|
| ITPRIPL1 | 4.373 | 0 |
| B3GAT1 | 4.258 | 0 |
| MMP23B | 4.061 | 0 |
| PPP2R2B | 4.058 | 0 |
| KLRD1 | 3.826 | 0 |
| GPR56 | 3.780 | 0 |
| PLEKHF1 | 3.748 | 0 |
| LAG3 | 3.644 | 0 |
| CA6 | -3.654 | 0 |
| GAL3ST4 | -3.552 | 0 |
| HAR1B | -3.537 | 0 |

+8 / -3
