# Supplementary material for: Phenotype and molecular signature of CD8+ T cell subsets in T cell- mediated rejections after kidney transplantation
Source: PLoS One. 2020 Jun 12;15(6):e0234323. doi: 10.1371/journal.pone.0234323 (PMC7292394; doi:10.1371/journal.pone.0234323)
Supplement: S3 Table — (PDF) [file pone.0234323.s004.pdf]

**Supplementary Table 3. Significantly changed genes along *ex vivo* $CD28^{null}CD57^{+}CD8^{+}$T cells**

| Gene Symbol | Score (d) | q-value (%) |
|---|---|---|
| ITPRIPL1 | 4.672 | 0 |
| LAG3 | 4.622 | 0 |
| CCDC157 | 4.543 | 0 |
| MMP23B | 4.410 | 0 |
| MATK | 4.320 | 0 |
| TARP | 4.301 | 0 |
| B3GAT1 | 4.216 | 0 |
| CLIC3 | 4.075 | 0 |
| PRAMEF11 | 4.064 | 0 |
| RGNEF | 4.034 | 0 |
| CEND1 | 3.983 | 0 |
| RAB11FIP5 | 3.895 | 0 |
| OR10H2 | 3.771 | 0 |
| TTC16 | 3.751 | 0 |
| ATP9A | 3.692 | 0 |
| ABCB5 | 3.671 | 0 |
| ABI3 | 3.660 | 0 |
| KIF19 | 3.660 | 0 |
| NBEAL2 | 3.647 | 0 |
| GPR56 | 3.633 | 0 |
| MAPT | 3.583 | 0 |
| CHST13 | 3.575 | 0 |
| BDKRB1 | 3.559 | 0 |
| OR7E156P | 3.542 | 0 |
| CD8A | 3.533 | 0 |
| TMEM179 | 3.487 | 2.46 |
| CACNA2D2 | 3.480 | 2.46 |
| PPP2R2B | 3.476 | 2.46 |
| Q93YZ4 | 3.461 | 2.46 |
| ATP6AP1L | 3.455 | 2.46 |
| C1orf21 | 3.439 | 2.46 |
| TPTE2 | 3.435 | 2.46 |
| TBX1 | 3.430 | 2.46 |
| KLHL35 | 3.420 | 2.46 |
| KLRD1 | 3.419 | 2.46 |
| ASCL2 | 3.372 | 2.46 |
| PASD1 | 3.372 | 2.46 |

| | | |
|---|---|---|
| GPX1 | 3.365 | 2.46 |
| C19orf12 | 3.349 | 2.46 |
| NPTX1 | 3.340 | 2.46 |
| RAPSN | 3.336 | 2.46 |
| C12orf75 | 3.332 | 2.46 |
| EVPLL | 3.331 | 2.46 |
| HES6 | 3.317 | 2.46 |
| FLRT3 | 3.314 | 2.46 |
| RDH13 | 3.309 | 2.46 |
| PDGFRB | 3.307 | 2.46 |
| C19orf25 | 3.296 | 2.46 |
| LIMK1 | 3.295 | 2.46 |
| ABCA3 | 3.292 | 2.46 |
| SEC14L3 | 3.290 | 2.46 |
| SMAD7 | 3.273 | 2.46 |
| FLJ46020 | 3.273 | 2.46 |
| ITGAL | 3.270 | 2.46 |
| FOXD1 | 3.269 | 2.46 |
| NAMA | 3.267 | 2.46 |
| OR1S1 | 3.267 | 2.46 |
| NTHL1 | 3.242 | 2.46 |
| CD27 | 3.239 | 2.46 |
| PLAC1 | 3.233 | 2.46 |
| NAPA | 3.227 | 2.46 |
| ACTL7A | 3.223 | 2.46 |
| MYH10 | 3.223 | 2.46 |
| ZDHHC24 | 3.202 | 2.46 |
| ATF3 | 3.196 | 2.46 |
| IRX4 | 3.168 | 2.46 |
| MB21D1 | 3.167 | 2.46 |
| ZNF333 | 3.160 | 2.46 |
| GPAA1 | 3.158 | 2.46 |
| MRGPRG | 3.154 | 2.46 |
| FGF17 | 3.153 | 2.46 |
| ZBTB3 | 3.150 | 2.46 |
| ICAM5 | 3.144 | 2.46 |
| CD81 | 3.135 | 2.46 |
| CEP78 | 3.133 | 2.46 |
| AHRR | 3.123 | 2.46 |

| | | |
|---|---|---|
| C19orf22 | 3.120 | 2.46 |
| TFDP2 | 3.108 | 2.46 |
| CST7 | 3.106 | 2.46 |
| TM4SF19 | 3.088 | 2.46 |
| DPP3 | 3.084 | 2.46 |
| ESCO2 | 3.080 | 2.46 |
| CD70 | 3.079 | 2.46 |
| CASZ1 | 3.076 | 2.46 |
| DDX49 | 3.066 | 2.46 |
| MIR205HG | 3.063 | 2.46 |
| AMOTL1 | 3.058 | 2.46 |
| B4GALT7 | 3.058 | 2.46 |
| GNLY | 3.055 | 2.46 |
| BOK | 3.051 | 2.46 |
| IL17REL | 3.048 | 2.46 |
| MUC8 | 3.048 | 2.46 |
| OR7E12P | 3.042 | 2.46 |
| OPRD1 | 3.040 | 2.46 |
| PLEKHF1 | 3.036 | 2.46 |
| TMEM95 | 3.036 | 2.46 |
| TNFRSF11B | 3.036 | 2.46 |
| C16orf45 | 3.034 | 2.46 |
| SEMA7A | 3.028 | 2.46 |
| SPEM1 | 3.024 | 2.46 |
| GFRA3 | 3.021 | 2.46 |
| OR7E14P | 3.015 | 2.46 |
| PRIMA1 | 3.014 | 2.46 |
| PYROXD2 | 3.013 | 2.46 |
| GALK1 | 3.008 | 2.46 |
| BEAN1 | 3.005 | 2.46 |
| MYO1G | 3.004 | 2.46 |
| FOXH1 | 3.000 | 2.46 |
| HOXA11-AS1 | 2.999 | 2.46 |
| WNT4 | 2.992 | 2.46 |
| RBPMS | 2.990 | 2.46 |
| CHAT | 2.987 | 2.46 |
| ADARB2-AS1 | 2.986 | 2.46 |
| MT1E | 2.983 | 2.46 |
| FSCN2 | 2.982 | 2.46 |

| | | |
|---|---|---|
| FLJ34208 | 2.968 | 2.46 |
| PDZRN3 | 2.967 | 2.46 |
| TBC1D7 | 2.967 | 2.46 |
| ADM2 | 2.966 | 2.46 |
| BAHCC1 | 2.964 | 2.46 |
| KCNK1 | 2.962 | 2.46 |
| INSL3 | 2.962 | 2.46 |
| PDIA2 | 2.960 | 2.46 |
| OR4K14 | 2.959 | 2.46 |
| PLAG1 | -4.151 | 0 |
| GAL3ST4 | -4.070 | 0 |
| PLCL1 | -4.010 | 0 |
| FAM134B | -3.679 | 2.46 |
| EFHC2 | -3.585 | 2.46 |
| LEF1 | -3.543 | 2.46 |
| KRTAP5-6 | -3.510 | 2.46 |
| ZNF540 | -3.451 | 2.46 |
| PKIA | -3.420 | 2.46 |
| NOG | -3.418 | 2.46 |
| FAM153A | -3.412 | 2.46 |
| FOXO1 | -3.403 | 2.46 |
| C2orf89 | -3.400 | 2.46 |
| ZNF471 | -3.391 | 2.46 |
| PDK1 | -3.341 | 2.46 |
| CEP41 | -3.338 | 2.46 |
| FBLN5 | -3.332 | 2.46 |
| MEST | -3.324 | 2.46 |
| C2orf40 | -3.304 | 2.46 |

+ 124/ -19
