# Supplementary material for: Phenotype and molecular signature of CD8+ T cell subsets in T cell- mediated rejections after kidney transplantation
Source: PLoS One. 2020 Jun 12;15(6):e0234323. doi: 10.1371/journal.pone.0234323 (PMC7292394; doi:10.1371/journal.pone.0234323)
Supplement: S4 Table — (PDF) [file pone.0234323.s005.pdf]

Supplementary Table 4. Up-regulated genes in $CCR7^+CD8^+$ T cells compared with $CCR7^-CD8^+$ T cells.

Gene Accession	Gene_Symbol	Gene Description	FC
NM_001301714	CCR7	chemokine (C-C motif) receptor 7	23.5
NM_002371	MAL	mal, T-cell differentiation protein	21.9
NM_001099658	LRRN3	leucine rich repeat neuronal 3	19.1
NM_001037535	SCML1	sex comb on midleg-like 1 (Drosophila)	14.2
NM_001130713	LEF1	lymphoid enhancer-binding factor 1	13.0
NM_020404	CD248	CD248 molecule, endosialin	12.9
ENST00000360669	FAM153A	family with sequence similarity 153, member A	11.1
NM_001145107	NELL2	neural EGFL like 2	9.8
NM_001293274	CACHD1	cache domain containing 1	8.0
NM_004948	DSC1	desmocollin 1	6.8
NM_001102	ACTN1	actinin, alpha 1	6.8
NR_029373	LEF1-AS1	LEF1 antisense RNA 1	6.6
NM_016591	GCNT4	glucosaminyl (N-acetyl) transferase 4, core 2	6.1
NM_001037132	NRCAM	neuronal cell adhesion molecule	6.1
NM_001170794	BACH2	BTB and CNC homology 1, basic leucine zipper transcription factor 2	5.8
NM_001174071	SERINC5	serine incorporator 5	5.8
NM_001190981	IL6ST	interleukin 6 signal transducer	5.7
NM_000565	IL6R	interleukin 6 receptor	5.3
NM_001173977	LRRC16A	leucine rich repeat containing 16A	5.1
NM_001251977	RCAN3	RCAN family member 3	5.0
NM_001256482	EPHX2	epoxide hydrolase 2, cytoplasmic	4.9
NM_003498	SNN	stannin	4.9
NM_003196	TCEA3	transcription elongation factor A (SII), 3	4.8
NM_001168647	CNKSR2	connector enhancer of kinase suppressor of Ras 2	4.8

NM_002737	PRKCA	protein kinase C, alpha	4.6
NM_001256850	TTN	titin	4.6
NR_103776	CHRM3-AS2	CHRM3 antisense RNA 2	4.5
NM_001080824	TRABD2A	TraB domain containing 2A	4.5
NM_001289010	MAN1C1	mannosidase, alpha, class 1C, member 1	4.5
NM_032160	DSEL	dermatan sulfate epimerase-like	4.3
NM_024637	GAL3ST4	galactose-3-O-sulfotransferase 4	4.3
NM_001170553	VSIG1	V-set and immunoglobulin domain containing 1	4.1
NM_173511	FAM117B	family with sequence similarity 117, member B	4.1
NM_003737	DCHS1	dachsous cadherin-related 1	4.1
NM_001243254	DENND5A	DENN/MADD domain containing 5A	4.0
NM_001204813	NT5E	5-nucleotidase, ecto (CD73)	4.0
NM_001287005	SUSD3	sushi domain containing 3	3.9
NM_031469	SH3BGRL2	SH3 domain binding glutamate-rich protein like 2	3.9
NR_036521	ZNF667-AS1	ZNF667 antisense RNA 1 (head to head)	3.9
NM_032427	MAML2	mastermind-like transcriptional coactivator 2	3.9
NM_001278549	PDK1	pyruvate dehydrogenase kinase, isozyme 1	3.8
NM_001270942	KLF7	Kruppel-like factor 7 (ubiquitous)	3.8
NM_001144952	SDK2	sidekick cell adhesion molecule 2	3.7
OTTHUMT00000410945	TRAJ53	T cell receptor alpha joining 53	3.6
NR_045407	RCAN3AS	RCAN3 antisense	3.6
NM_018593	SLC16A10	solute carrier family 16 (aromatic amino acid transporter), member 10	3.6
NM_001060	TBXA2R	thromboxane A2 receptor	3.5
OTTHUMT00000351233	TRBV9	T cell receptor beta variable 9	3.5

NM_001035254	FAM102A	family with sequence similarity 102, member A	3.5
NM_016224	SNX9	sorting nexin 9	3.5
NM_001215	CA6	carbonic anhydrase VI	3.4
NM_001267036	SLC7A8	solute carrier family 7 (amino acid transporter light chain, L system), member 8	3.4
NM_001098815	TESPA1	thymocyte expressed, positive selection associated 1	3.3
NM_003863	DPM2	dolichyl-phosphate mannosyltransferase polypeptide 2, regulatory subunit	3.3
NM_005450	NOG	noggin	3.3
XM_011520383	ATHL1	ATH1, acid trehalase-like 1 (yeast)	3.3
NM_000655	SELL	selectin L	3.3
NM_001316745	CCDC141	coiled-coil domain containing 141	3.2
NM_021095	SLC5A6	solute carrier family 5 (sodium/multivitamin and iodide cotransporter), member 6	3.2
NM_001114634	PLAG1	pleiomorphic adenoma gene 1	3.2
NM_130446	KLHL6	kelch-like family member 6	3.2
NM_001134851	TCF7	transcription factor 7 (T-cell specific, HMG-box)	3.2
NR_029832	MIR29C	microRNA 29c	3.2
NM_022336	EDAR	ectodysplasin A receptor	3.1
NM_001076785	SLC7A6	solute carrier family 7 (amino acid transporter light chain, y+L system), member 6	3.1
ENST00000586713	CEP295NL	CEP295 N-terminal like	3.1
NM_001307952	HAPLN3	hyaluronan and proteoglycan link protein 3	3.1
NM_001172435	RAB3GAP1	RAB3 GTPase activating protein subunit 1 (catalytic)	3.1
NM_001256126	CERS6	ceramide synthase 6	3.1
NM_001145426	YBX3	Y box binding protein 3	3.1
NM_001080437	SNED1	sushi, nidogen and EGF-like domains 1	3.1
NR_103784	ITPKB-IT1	ITPKB intronic transcript 1	3.1

OTTHUMT00000410959	TRAJ39	T cell receptor alpha joining 39	3.1
NM_001256541	TMEM204	transmembrane protein 204	3.1
NM_001271856	GRASP	GRP1 (general receptor for phosphoinositides 1)-associated scaffold protein	3.1
NM_001265615	FAM153B	family with sequence similarity 153, member B	3.0
NM_001006658	CR2	complement component (3d/Epstein Barr virus) receptor 2	3.0
NM_203411	TMEM88	transmembrane protein 88	3.0
NM_015888	HOOK1	hook microtubule-tethering protein 1	3.0
NM_001130172	MYB	v-myb avian myeloblastosis viral oncogene homolog	3.0
NM_052847	GNG7	guanine nucleotide binding protein (G protein), gamma 7	3.0
NM_001271594	SESN3	sestrin 3	3.0
NM_001260489	LSR	lipolysis stimulated lipoprotein receptor	2.9
NM_152272	CHMP7	charged multivesicular body protein 7	2.9
NM_000922	PDE3B	phosphodiesterase 3B, cGMP-inhibited	2.9
ENST00000532740	PDE3B	phosphodiesterase 3B, cGMP-inhibited	2.9
NM_001033045	GPR155	G protein-coupled receptor 155	2.9
NM_002518	NPAS2	neuronal PAS domain protein 2	2.9
NM_001003407	ABLIM1	actin binding LIM protein 1	2.9
NM_138381	OXNAD1	oxidoreductase NAD-binding domain containing 1	2.8
NR_109930	CTD-2201I18.1	uncharacterized LOC101929215	2.8
NM_012093	AK5	adenylate kinase 5	2.8
NM_015394	ZNF10	zinc finger protein 10	2.8
NM_001345	DGKA	diacylglycerol kinase alpha	2.8
NR_039641	MIR4439	microRNA 4439	2.8

NM_000875	IGF1R	insulin-like growth factor 1 receptor	2.8
NR_023382	ZNF815P	zinc finger protein 815, pseudogene	2.8
NM_144770	RBM11	RNA binding motif protein 11	2.8
NM_003447	ZNF165	zinc finger protein 165	2.8
NR_036502	PRKCQ-AS1	PRKCQ antisense RNA 1	2.8
NM_001012505	FOXP1	forkhead box P1	2.8
NM_001190259	GCSAM	germinal center-associated, signaling and motility	2.8
NM_006909	RASGRF2	Ras protein-specific guanine nucleotide-releasing factor 2	2.7
NM_006613	GRAP	GRB2-related adaptor protein	2.7
NM_001039508	SIRPG	signal-regulatory protein gamma	2.7
NM_001079906	ZNF331	zinc finger protein 331	2.7
NM_014059	RGCC	regulator of cell cycle	2.7
NM_001034850	FAM134B	family with sequence similarity 134, member B	2.7
NM_001204197	NBEA	neurobeachin	2.7
NR_033897	EPHA1-AS1	EPHA1 antisense RNA 1	2.7
NM_001164443	ANKRD31	ankyrin repeat domain 31	2.7
NM_003711	PLPP1	phospholipid phosphatase 1	2.7
NM_004326	BCL9	B-cell CLL/lymphoma 9	2.7
NM_007079	PTP4A3	protein tyrosine phosphatase type IVA, member 3	2.7
NM_001005339	RGS10	regulator of G-protein signaling 10	2.7
NM_212559	XKRX	X-linked Kx blood group related, X-linked	2.7
NM_030571	NDFIP1	Nedd4 family interacting protein 1	2.6
NM_000218	KCNQ1	potassium channel, voltage gated KQT-like subfamily Q, member 1	2.6
NM_005232	EPHA1	EPH receptor A1	2.6
NM_001130414	APBA2	amyloid beta (A4) precursor protein-binding, family A, member 2	2.6

NM_025092	ATHL1	ATH1, acid trehalase-like 1 (yeast)	2.6
OTTHUMT00000410953	TRAJ45	T cell receptor alpha joining 45	2.6
NR_037931	ANAPC1P1	anaphase promoting complex subunit 1 pseudogene 1	2.6
NM_000210	ITGA6	integrin alpha 6	2.6
XM_011517768	GLIS3	GLIS family zinc finger 3	2.6
XM_006710960	LRRC8C	leucine rich repeat containing 8 family, member C	2.6
OTTHUMT00000371928	FAM153B	family with sequence similarity 153, member B	2.6
NM_001783	CD79A	CD79a molecule, immunoglobulin-associated alpha	2.6
NM_006931	SLC2A3	solute carrier family 2 (facilitated glucose transporter), member 3	2.6
NM_001204204	SEC14L2	SEC14-like lipid binding 2	2.6
NM_001164716	PYGM	phosphorylase, glycogen, muscle	2.6
NM_002072	GNAQ	guanine nucleotide binding protein (G protein), q polypeptide	2.6
NR_003317	SNORD116-2	small nucleolar RNA, C/D box 116-2	2.5
NM_006272	S100B	S100 calcium binding protein B	2.5
NM_014938	MLXIP	MLX interacting protein	2.5
NM_003890	FCGBP	Fc fragment of IgG binding protein	2.5
NM_024758	AGMAT	agmatinase	2.5
ENST00000423733	PLCG1	phospholipase C, gamma 1	2.5
NM_000574	CD55	CD55 molecule, decay accelerating factor for complement (Cromer blood group)	2.5
NM_019072	SGTB	small glutamine-rich tetratricopeptide repeat (TPR)-containing, beta	2.5
NM_015351	TTC9	tetratricopeptide repeat domain 9	2.5

NM_001143819	TPCN1	two pore segment channel 1	2.5
NM_001253900	MEST	mesoderm specific transcript	2.5
NM_001309242	MYO15B	myosin XVB	2.5
NM_001236	CBR3	carbonyl reductase 3	2.5
NM_001145845	ROBO1	roundabout guidance receptor 1	2.5
NM_005164	ABCD2	ATP binding cassette subfamily D member 2	2.5
NM_001085384	ZNF154	zinc finger protein 154	2.5
NM_001277291	ZNF891	zinc finger protein 891	2.5
NM_001032221	STXBP1	syntaxin binding protein 1	2.5
NM_005567	LGALS3BP	lectin, galactoside-binding, soluble, 3 binding protein	2.5
NM_001001567	PDE9A	phosphodiesterase 9A	2.5
NM_001190482	PCSK5	proprotein convertase subtilisin/kexin type 5	2.5
NM_001136180	HSBP1L1	heat shock factor binding protein 1-like 1	2.5
NM_003749	IRS2	insulin receptor substrate 2	2.5
NR_003361	SNORD116-28	small nucleolar RNA, C/D box 116-28	2.5
NM_003328	TXK	TXK tyrosine kinase	2.5
NM_006493	CLN5	ceroid-lipofuscinosis, neuronal 5	2.4
NR_003321	SNORD116-6	small nucleolar RNA, C/D box 116-6	2.4
NR_030353	MIR623	microRNA 623	2.4
OTTHUMT00000410944	TRAJ54	T cell receptor alpha joining 54	2.4
NM_001281429	PCED1B	PC-esterase domain containing 1B	2.4
NR_029506	MIR32	microRNA 32	2.4
NM_052916	RNF157	ring finger protein 157	2.4
NM_207360	ZC3H12D	zinc finger CCCH-type containing 12D	2.4
XM_011519416	ANKRD26	ankyrin repeat domain 26	2.4

ENST00000488284	RASA4B	RAS p21 protein activator 4B	2.4
NM_001278359	RHOH	ras homolog family member H	2.4
NM_024959	SLC8B1	solute carrier family 8 (sodium/lithium/calcium exchanger), member B1	2.4
NR_003330	SNORD116-15	small nucleolar RNA, C/D box 116-15	2.4
NR_037443	MIR3671	microRNA 3671	2.4
NM_001277	CHKA	choline kinase alpha	2.4
NM_002467	MYC	v-myc avian myelocytomatosis viral oncogene homolog	2.4
NM_001165412	NFKB1	nuclear factor of kappa light polypeptide gene enhancer in B-cells 1	2.4
NM_001744	CAMK4	calcium/calmodulin-dependent protein kinase IV	2.4
NM_020741	KIAA1257	KIAA1257	2.4
NM_001135911	PIK3IP1	phosphoinositide-3-kinase interacting protein 1	2.4
NM_015627	LDLRAP1	low density lipoprotein receptor adaptor protein 1	2.3
NM_001102406	ALPK1	alpha kinase 1	2.3
NM_001242	CD27	CD27 molecule	2.3
BX647165	TRAV20	T cell receptor alpha variable 20	2.3
NM_001204502	FLT3LG	fms-related tyrosine kinase 3 ligand	2.3
NM_001286247	RALGPS2	Ral GEF with PH domain and SH3 binding motif 2	2.3
NM_032184	SYDE2	synapse defective 1, Rho GTPase, homolog 2 (C. elegans)	2.3
NM_001083910	SIRPB1	signal-regulatory protein beta 1	2.3
OTTHUMT00000401893	TRAV8-4	T cell receptor alpha variable 8-4	2.3
NM_001135109	RIC3	RIC3 acetylcholine receptor chaperone	2.3
NM_178563	AGBL3	ATP/GTP binding protein-like 3	2.3
NM_001099410	GPRASP1	G protein-coupled receptor associated sorting protein 1	2.3

NM_001134476	LRRC8B	leucine rich repeat containing 8 family, member B	2.3
NR_002982	SNORA54	small nucleolar RNA, H/ACA box 54	2.3
NR_110542	PIK3IP1-AS1	PIK3IP1 antisense RNA 1 (head to head)	2.3
NM_001085357	BTLA	B and T lymphocyte associated	2.3
NM_001145304	KIAA1683	KIAA1683	2.3
NM_001005328	OR2A7	olfactory receptor, family 2, subfamily A, member 7	2.3
NM_001164317	FLNB	filamin B, beta	2.3
NM_001170755	NBPF15	neuroblastoma breakpoint family, member 15	2.3
NM_173354	SIK1	salt-inducible kinase 1	2.2
NM_001114614	MFGE8	milk fat globule-EGF factor 8 protein	2.2
NM_024315	TMEM243	transmembrane protein 243, mitochondrial	2.2
NM_001276318	PPP1R3E	protein phosphatase 1, regulatory subunit 3E	2.2
NR_001290	SNORD116-19	small nucleolar RNA, C/D box 116-19	2.2
NR_001290	SNORD116-19	small nucleolar RNA, C/D box 116-19	2.2
NM_001128208	LEPROTL1	leptin receptor overlapping transcript-like 1	2.2
NM_001282674	NGFRAP1	nerve growth factor receptor (TNFRSF16) associated protein 1	2.2
AF400486	SNRPN	small nuclear ribonucleoprotein polypeptide N	2.2
NM_001110798	NAA16	N(alpha)-acetyltransferase 16, NatA auxiliary subunit	2.2
NR_030398	MIR421	microRNA 421	2.2
NM_001252119	PASK	PAS domain containing serine/threonine kinase	2.2
XM_011516411	PRSS1	protease, serine, 1 (trypsin 1)	2.2
OTTHUMT00000410946	TRAJ52	T cell receptor alpha joining 52	2.2
NM_001100812	CXCL16	chemokine (C-X-C motif) ligand 16	2.2
NM_001129778	GRAPL	GRB2-related adaptor protein-like	2.2

NM_021915	ZNF69	zinc finger protein 69	2.2
NR_026932	PDCD4-AS1	PDCD4 antisense RNA 1	2.2
AF400486	SNRPN	small nuclear ribonucleoprotein polypeptide N	2.2
NM_001251882	VIPR1	vasoactive intestinal peptide receptor 1	2.2
NM_001136501	ZNF844	zinc finger protein 844	2.2
NM_001135648	PTPRK	protein tyrosine phosphatase, receptor type, K	2.2
NM_001098623	OBSCN	obscurin, cytoskeletal calmodulin and titin-interacting RhoGEF	2.2
NM_004915	ABCG1	ATP binding cassette subfamily G member 1	2.2
NR_003047	SNORD19	small nucleolar RNA, C/D box 19	2.2
NM_001128931	NAF1	nuclear assembly factor 1 ribonucleoprotein	2.2
NR_108047	RNF139-AS1	RNF139 antisense RNA 1 (head to head)	2.2
ENST00000558190	ATXN3	ataxin 3	2.2
NM_019050	USP53	ubiquitin specific peptidase 53	2.1
OTTHUMT00000409911	TRAV26-2	T cell receptor alpha variable 26-2	2.1
NM_002221	ITPKB	inositol-trisphosphate 3-kinase B	2.1
NM_003840	TNFRSF10D	tumor necrosis factor receptor superfamily, member 10d, decoy with truncated death domain	2.1
NM_001097622	OCM	oncomodulin	2.1
XM_006723698	ZNF831	zinc finger protein 831	2.1
NM_001005218	OR5B21	olfactory receptor, family 5, subfamily B, member 21	2.1
AL832294	ARHGEF1	Rho guanine nucleotide exchange factor 1	2.1
NM_001289050	SLC22A17	solute carrier family 22, member 17	2.1
NM_001300735	RASGEF1B	RasGEF domain family member 1B	2.1
NM_005534	IFNGR2	interferon gamma receptor 2 (interferon gamma transducer 1)	2.1
NM_006823	PKIA	protein kinase (cAMP-dependent, catalytic) inhibitor alpha	2.1

NM_022117	TSPYL2	TSPY-like 2	2.1
NM_152653	UBE2E2	ubiquitin conjugating enzyme E2E 2	2.1
NM_177437	TAS2R60	taste receptor, type 2, member 60	2.1
AF400486	SNRPN	small nuclear ribonucleoprotein polypeptide N	2.1
NM_002114	HIVEP1	human immunodeficiency virus type I enhancer binding protein 1	2.1
NM_053055	THEM4	thioesterase superfamily member 4	2.1
NR_002157	OR2A9P	olfactory receptor, family 2, subfamily A, member 9 pseudogene	2.1
NM_001010974	BCAS4	breast carcinoma amplified sequence 4	2.1
NR_037507	MIR3942	microRNA 3942	2.1
NM_001306080	LMO7	LIM domain 7	2.1
NM_017918	CCDC109B	coiled-coil domain containing 109B	2.1
NM_001256732	SSBP2	single-stranded DNA binding protein 2	2.1
NM_001244	TNFSF8	tumor necrosis factor (ligand) superfamily, member 8	2.1
NM_001297707	CAMSAP2	calmodulin regulated spectrin-associated protein family, member 2	2.1
NM_015147	CEP68	centrosomal protein 68kDa	2.1
NM_001099289	SH3RF3	SH3 domain containing ring finger 3	2.1
NM_001657	AREG	amphiregulin	2.1
NM_001131010	SATB1	SATB homeobox 1	2.1
NR_003697	SNHG15	small nucleolar RNA host gene 15	2.1
NM_015042	ZNF609	zinc finger protein 609	2.1
NM_020651	PELI1	pellino E3 ubiquitin protein ligase 1	2.1
NM_001081	CUBN	cubilin (intrinsic factor-cobalamin receptor)	2.1
NM_012092	ICOS	inducible T-cell co-stimulator	2.1
NM_004625	WNT7A	wingless-type MMTV integration site family, member 7A	2.0

NM_001024847	TGFBR2	transforming growth factor beta receptor II	2.0
ENST00000511560	AREG	amphiregulin	2.0
NM_001171137	STRBP	spermatid perinuclear RNA binding protein	2.0
NR_036191	MIR4306	microRNA 4306	2.0
OTTHUMT00000410951	TRAJ47	T cell receptor alpha joining 47	2.0
NM_001080424	KDM6B	lysine (K)-specific demethylase 6B	2.0
NM_001300949	ZNF101	zinc finger protein 101	2.0
NM_014784	ARHGEF11	Rho guanine nucleotide exchange factor 11	2.0
AK095849	SNHG1	small nucleolar RNA host gene 1	2.0
NM_001104595	FAM118A	family with sequence similarity 118, member A	2.0
NM_032873	UBASH3B	ubiquitin associated and SH3 domain containing B	2.0
NM_001004313	TMEM220	transmembrane protein 220	2.0
NM_001304458	PRMT9	protein arginine methyltransferase 9	2.0
NM_001012957	DISC1	disrupted in schizophrenia 1	2.0
NM_001242318	PDE7A	phosphodiesterase 7A	2.0
NM_001135564	HSF2	heat shock transcription factor 2	2.0
NM_001257194	KLHL3	kelch-like family member 3	2.0
NM_000633	BCL2	B-cell CLL/lymphoma 2	2.0
OTTHUMT00000409906	TRAV29DV5	T cell receptor alpha variable 29/delta variable 5 (gene/pseudogene)	2.0
NR_038353	FAM153C	family with sequence similarity 153, member C, pseudogene	2.0
NM_022552	DNMT3A	DNA (cytosine-5-)-methyltransferase 3 alpha	2.0
NM_001307977	WDR74	WD repeat domain 74	2.0
NM_001031714	INF2	inverted formin, FH2 and WH2 domain containing	2.0
NM_020448	NIPAL3	NIPA-like domain containing 3	2.0
NM_001172303	MASTL	microtubule associated serine/threonine kinase-like	2.0
NR_022008	PWAR5	Prader Willi/Angelman region RNA 5	2.0

NR_022011	PWARSN	Prader Willi/Angelman region RNA, SNRPN neighbor	2.0
NM_001114171	FOSB	FBJ murine osteosarcoma viral oncogene homolog B	2.0
NM_016148	SHANK1	SH3 and multiple ankyrin repeat domains 1	2.0
NM_022355	DPEP2	dipeptidase 2	2.0
NM_001284239	SARAF	store-operated calcium entry-associated regulatory factor	2.0
NM_173602	DIP2B	disco-interacting protein 2 homolog B	2.0
AF400486	SNRPN	small nuclear ribonucleoprotein polypeptide N	2.0
NM_001079524	PAICS	phosphoribosylaminoimidazole carboxylase, phosphoribosylaminoimidazole succinocarboxamide synthetase	1.9
NM_001008530	LGMN	legumain	1.9
NM_001042413	GLIS3	GLIS family zinc finger 3	1.9
NM_000070	CAPN3	calpain 3	1.9
NR_003057	SNORD69	small nucleolar RNA, C/D box 69	1.9
NM_001193270	MSL3	male-specific lethal 3 homolog (Drosophila)	1.9
OTTHUMT00000410976	TRAJ22	T cell receptor alpha joining 22	1.9
NR_029616	MIR187	microRNA 187	1.9
NM_003596	TPST1	tyrosylprotein sulfotransferase 1	1.9
NM_001098412	GAGE13	G antigen 13	1.9
NR_002923	BCORP1	BCL6 corepressor pseudogene 1	1.9
NM_002167	ID3	inhibitor of DNA binding 3, dominant negative helix-loop-helix protein	1.9
NM_001042574	CRTC3	CREB regulated transcription coactivator 3	1.9
NM_005539	INPP5A	inositol polyphosphate-5-phosphatase A	1.9
AF400486	SNRPN	small nuclear ribonucleoprotein polypeptide N	1.9
NR_030364	MIR634	microRNA 634	1.9
NM_001144034	TMEM25	transmembrane protein 25	1.9

NM_001164	APBB1	amyloid beta (A4) precursor protein-binding, family B, member 1 (Fe65)	1.9
NM_001202233	NR4A1	nuclear receptor subfamily 4, group A, member 1	1.9
NM_001174097	LDHB	lactate dehydrogenase B	1.9
NM_001105539	ZBTB10	zinc finger and BTB domain containing 10	1.9
NM_014698	TMEM63A	transmembrane protein 63A	1.9
NM_001146094	FAN1	FANCD2/FANCI-associated nuclease 1	1.9
OTTHUMT00000410954	TRAJ44	T cell receptor alpha joining 44	1.9
NM_001277335	RASA4B	RAS p21 protein activator 4B	1.9
XM_011529753	CHAF1B	chromatin assembly factor 1, subunit B (p60)	1.9
NM_006578	GNB5	guanine nucleotide binding protein (G protein), beta 5	1.9
NM_001287036	KIAA1958	KIAA1958	1.9
NM_001005287	OR2A1	olfactory receptor, family 2, subfamily A, member 1	1.9
NM_000901	NR3C2	nuclear receptor subfamily 3, group C, member 2	1.9
NM_001171818	PPARD	peroxisome proliferator-activated receptor delta	1.9
OTTHUMT00000401872	TRAV1-1	T cell receptor alpha variable 1-1	1.9
XM_006711826	OBSCN	obscurin, cytoskeletal calmodulin and titin-interacting RhoGEF	1.9
NM_004073	PLK3	polo-like kinase 3	1.9
NM_015041	CLUAP1	clusterin associated protein 1	1.9
NM_005646	TARBP1	TAR (HIV-1) RNA binding protein 1	1.9
NM_006598	SLC12A7	solute carrier family 12 (potassium/chloride transporter), member 7	1.9
AK057897	CRHR1-IT1	CRHR1 intronic transcript 1	1.9

NR_038286	PSMD6-AS2	PSMD6 antisense RNA 2	1.9
NM_176877	INADL	InaD-like (Drosophila)	1.9
NM_001166103	SPINT2	serine peptidase inhibitor, Kunitz type, 2	1.9
NM_020451	SEPN1	selenoprotein N, 1	1.9
NM_001178100	CD8B	CD8b molecule	1.9
NR_002952	SNORA9	small nucleolar RNA, H/ACA box 9	1.9
NM_001301054	PCYOX1L	prenylcysteine oxidase 1 like	1.9
AF400486	SNRPN	small nuclear ribonucleoprotein polypeptide N	1.9
NM_138435	FAM83F	family with sequence similarity 83, member F	1.9
NR_030299	MIR573	microRNA 573	1.9
ENST00000622469	ZNRD1-AS1	ZNRD1 antisense RNA 1	1.9
NM_001042494	SLC12A6	solute carrier family 12 (potassium/chloride transporter), member 6	1.9
NM_001164114	CASS4	Cas scaffolding protein family member 4	1.9
NM_002568	PABPC1	poly(A) binding protein, cytoplasmic 1	1.9
ENST00000247815	HELB	helicase (DNA) B	1.9
NM_080603	ZSWIM1	zinc finger, SWIM-type containing 1	1.9
NM_001199741	GADD45A	growth arrest and DNA-damage-inducible, alpha	1.9
NR_039778	MIR4635	microRNA 4635	1.8
NM_013240	N6AMT1	N-6 adenine-specific DNA methyltransferase 1 (putative)	1.8
NM_001277285	IGSF9B	immunoglobulin superfamily, member 9B	1.8

ENST00000473426	ARHGAP15	Rho GTPase activating protein 15	1.8
ENST00000285737	LONP2	lon peptidase 2, peroxisomal	1.8
NM_001164605	FXYD5	FXYD domain containing ion transport regulator 5	1.8
NM_001253875	UXS1	UDP-glucuronate decarboxylase 1	1.8
NR_033347	SNORA70E	small nucleolar RNA, H/ACA box 70E	1.8
NM_001013437	SEH1L	SEH1-like nucleoporin	1.8
NM_005546	ITK	IL2-inducible T-cell kinase	1.8
OTTHUMT00000410988	TRAJ10	T cell receptor alpha joining 10	1.8
NM_003463	PTP4A1	protein tyrosine phosphatase type IVA, member 1	1.8
NM_001136199	GRAMD1A	GRAM domain containing 1A	1.8
NM_001012398	AKTIP	AKT interacting protein	1.8
NM_001270960	NOSIP	nitric oxide synthase interacting protein	1.8
NM_024989	PGAP1	post-GPI attachment to proteins 1	1.8
AF319524	SNRPN	small nuclear ribonucleoprotein polypeptide N	1.8
NM_002229	JUNB	jun B proto-oncogene	1.8
NR_037498	MIR3934	microRNA 3934	1.8
AF400487	SNRPN	small nuclear ribonucleoprotein polypeptide N	1.8
NM_022754	SFXN1	sideroflexin 1	1.8
NM_001287529	MFSD12	major facilitator superfamily domain containing 12	1.8
NM_001198978	SMAP2	small ArfGAP2	1.8

XM_005250085	RASA4B	RAS p21 protein activator 4B	1.8
ENST00000504352	PCBD2	pterin-4 alpha-carbinolamine dehydratase/dimerization cofactor of hepatocyte nuclear factor 1 alpha (TCF1) 2	1.8
NR_003329	SNORD116-14	small nucleolar RNA, C/D box 116-14	1.8
NM_000276	OCRL	oculocerebrorenal syndrome of Lowe	1.8
NR_039824	MIR4677	microRNA 4677	1.8
NM_001024956	SC5D	sterol-C5-desaturase	1.8
NM_001002255	SUMO4	small ubiquitin-like modifier 4	1.8
NM_001004720	NCK2	NCK adaptor protein 2	1.8
NR_003584	SNHG8	small nucleolar RNA host gene 8	1.8
NM_020405	PLXDC1	plexin domain containing 1	1.8
OTTHUMT00000410994	TRAJ4	T cell receptor alpha joining 4	1.8
NM_001142784	IL11RA	interleukin 11 receptor, alpha	1.8
NM_001136537	BTBD19	BTB (POZ) domain containing 19	1.8
OTTHUMT00000401891	TRAV13-1	T cell receptor alpha variable 13-1	1.8
NR_003360	SNORD116-29	small nucleolar RNA, C/D box 116-29	1.8
NR_003326	SNORD116-11	small nucleolar RNA, C/D box 116-11	1.8
NM_001005404	YPEL2	yippee like 2	1.8
NM_015395	TECPR1	tectonin beta-propeller repeat containing 1	1.7
NM_012216	MID2	midline 2	1.7
NM_178026	GGT7	gamma-glutamyltransferase 7	1.7

NR_039696	MIR3689F	microRNA 3689f	1.7
OTTHUMT00000399953	SOD2	superoxide dismutase 2, mitochondrial	1.7
OTTHUMT00000401888	TRAV12-1	T cell receptor alpha variable 12-1	1.7
NR_003367	PVT1	Pvt1 oncogene (non-protein coding)	1.7
NM_003033	ST3GAL1	ST3 beta-galactoside alpha-2,3-sialyltransferase 1	1.7
NR_103741	IPO5P1	importin 5 pseudogene 1	1.7
NM_001304507	ZBTB25	zinc finger and BTB domain containing 25	1.7
AF086543	CD44	CD44 molecule (Indian blood group)	1.7
AF400486	SNRPN	small nuclear ribonucleoprotein polypeptide N	1.7
NM_002015	FOXO1	forkhead box O1	1.7
NM_014400	LYPD3	LY6/PLAUR domain containing 3	1.7
NR_024034	CLUHP3	clustered mitochondria (cluA/CLU1) homolog pseudogene 3	1.7
NM_001165258	TMEM14C	transmembrane protein 14C	1.7
NM_001142473	FCMR	Fc fragment of IgM receptor	1.7
NR_002327	SNORA10	small nucleolar RNA, H/ACA box 10	1.7
NM_001080425	BEX4	brain expressed X-linked 4	1.7
NM_001001895	UBASH3A	ubiquitin associated and SH3 domain containing A	1.7
NM_021078	KAT2A	K(lysine) acetyltransferase 2A	1.7
NM_001307924	NAP1L1	nucleosome assembly protein 1-like 1	1.7
NM_001127399	YPEL5	yippee like 5	1.7

NM_014611	MDN1	midasin AAA ATPase 1	1.7
NR_125803	SATB1-AS1	SATB1 antisense RNA 1	1.7
NM_152405	JMY	junction mediating and regulatory protein, p53 cofactor	1.7
NM_001130955	ARHGEF18	Rho/Rac guanine nucleotide exchange factor 18	1.7
NM_006981	NR4A3	nuclear receptor subfamily 4, group A, member 3	1.7
NM_001130048	DOCK9	dedicator of cytokinesis 9	1.7
XM_011530938	ZC3H12B	zinc finger CCCH-type containing 12B	1.7
ENST00000274242	RPL37	ribosomal protein L37	1.7
NM_001286631	RBM26	RNA binding motif protein 26	1.7
NM_001286262	TCP11L2	t-complex 11, testis-specific-like 2	1.7
NM_000418	IL4R	interleukin 4 receptor	1.7
NM_001142287	SEMA4D	sema domain, immunoglobulin domain (Ig), transmembrane domain (TM) and short cytoplasmic domain, (semaphorin) 4D	1.6
NM_003291	TPP2	tripeptidyl peptidase II	1.6
NM_001014436	DBNL	drebrin-like	1.6
NM_016584	IL23A	interleukin 23, alpha subunit p19	1.6
NM_001098200	GPR18	G protein-coupled receptor 18	1.6
NM_015986	CRLF3	cytokine receptor-like factor 3	1.6
NR_002779	NUDT9P1	nudix hydrolase 9 pseudogene 1	1.6
NR_037482	MIR3918	microRNA 3918	1.6
NM_001293735	LYRM7	LYR motif containing 7	1.6

NR_047032	SNORD116-30	small nucleolar RNA, C/D box 116-30	1.6
NR_046084	SH3BP5-AS1	SH3BP5 antisense RNA 1	1.6
NM_005252	FOS	FBJ murine osteosarcoma viral oncogene homolog	1.6
ENST00000610952	CD24	CD24 molecule	1.6
NM_014366	GNL3	guanine nucleotide binding protein-like 3 (nucleolar)	1.6
AK054610	SRSF1	serine/arginine-rich splicing factor 1	1.6
NR_039760	MIR378I	microRNA 378i	1.6
NR_046579	CACNA1C-AS2	CACNA1C antisense RNA 2	1.6
NM_017592	MED29	mediator complex subunit 29	1.6
NM_178868	CMTM8	CKLF-like MARVEL transmembrane domain containing 8	1.6
NM_001009	RPS5	ribosomal protein S5	1.6
OTTHUMT00000410964	TRAJ34	T cell receptor alpha joining 34	1.5
AF400486	SNRPN	small nuclear ribonucleoprotein polypeptide N	1.5
BC025727	TRAV20	T cell receptor alpha variable 20	1.5
NM_001285549	ZDBF2	zinc finger, DBF-type containing 2	1.5
NM_001004304	ZNF740	zinc finger protein 740	1.5
OTTHUMT00000351238	TRBV2	T cell receptor beta variable 2	1.5
NM_006186	NR4A2	nuclear receptor subfamily 4, group A, member 2	1.5
