# Supplementary material for: Phenotype and molecular signature of CD8+ T cell subsets in T cell- mediated rejections after kidney transplantation
Source: PLoS One. 2020 Jun 12;15(6):e0234323. doi: 10.1371/journal.pone.0234323 (PMC7292394; doi:10.1371/journal.pone.0234323)
Supplement: S5 Table — (PDF) [file pone.0234323.s006.pdf]

Supplementary Table 5. Down-regulated genes in CCR7^{+}CD8^{+} T cells compared with CCR7^{-}CD8^{+} T cells.

Gene Accession	Gene_Symbol	Gene Description	FC
NM_180991	SLCO4C1	solute carrier organic anion transporter family, member 4C1	**-19.0**
NM_001291822	KLRF1	killer cell lectin-like receptor subfamily F, member 1	**-17.4**
NM_002258	KLRB1	killer cell lectin-like receptor subfamily B, member 1	**-15.6**
NM_001270780	GZMH	granzyme H	**-15.5**
NM_001145770	ADGRG1	adhesion G protein-coupled receptor G1	**-13.9**
NM_053282	SH2D1B	SH2 domain containing 1B	**-13.7**
NM_003853	IL18RAP	interleukin 18 receptor accessory protein	**-12.5**
OTTHUMT00000410933	TRDJ4	T cell receptor delta joining 4	**-12.2**
OTTHUMT00000410936	TRDC	T cell receptor delta constant	**-12.1**
NM_031950	FGFBP2	fibroblast growth factor binding protein 2	**-10.7**
ENST00000628473	ZEB2	zinc finger E-box binding homeobox 2	**-10.6**
NM_144701	IL23R	interleukin 23 receptor	**-10.4**
NM_002288	LAIR2	leukocyte-associated immunoglobulin-like receptor 2	**-10.0**
NM_004131	GZMB	granzyme B	**-9.9**
NM_001081637	LILRB1	leukocyte immunoglobulin-like receptor, subfamily B (with TM and ITIM domains), member 1	**-9.8**
NM_002984	CCL4	chemokine (C-C motif) ligand 4	**-9.8**
NM_001114396	KLRD1	killer cell lectin-like receptor subfamily D, member 1	**-9.8**
NM_000024	ADRB2	adrenoceptor beta 2, surface	**-9.2**
NM_002661	PLCG2	phospholipase C, gamma 2 (phosphatidylinositol-specific)	**-9.2**
NM_005601	NKG7	natural killer cell granule protein 7	**-9.1**
NM_001136536	DTHD1	death domain containing 1	**-9.0**

NM_000632	ITGAM	integrin, alpha M (complement component 3 receptor 3 subunit)	**-8.7**
NM_003783	B3GALT2	UDP-Gal:betaGlcNAc beta 1,3-galactosyltransferase 2	**-8.7**
OTTHUMT00000410935	TRDJ3	T cell receptor delta joining 3	**-8.4**
NM_002664	PLEK	pleckstrin	**-8.4**
NM_001166215	S1PR5	sphingosine-1-phosphate receptor 5	**-8.3**
NM_006144	GZMA	granzyme A	**-7.7**
NM_013351	TBX21	T-box 21	**-7.4**
OTTHUMT00000410673	TRDJ1	T cell receptor delta joining 1	**-7.3**
NM_001083116	PRF1	perforin 1 (pore forming protein)	**-7.2**
NR_028092	LPAL2	lipoprotein, Lp(a)-like 2, pseudogene	**-7.1**
NM_001178015	SLC4A10	solute carrier family 4, sodium bicarbonate transporter, member 10	**-6.7**
NM_015103	PLXND1	plexin D1	**-6.6**
NM_030797	FAM49A	family with sequence similarity 49, member A	**-6.5**
BC072396	TRGV9	T cell receptor gamma variable 9	**-6.5**
NM_001031804	MAF	v-maf avian musculoaponeurotic fibrosarcoma oncogene homolog	**-6.5**
NM_001302758	GNLY	granulysin	**-6.5**
NM_001005505	CACNA2D2	calcium channel, voltage-dependent, alpha 2/delta subunit 2	**-6.4**
NM_001282588	SLAMF7	SLAM family member 7	**-6.4**
NM_015868	KIR2DL3	killer cell immunoglobulin-like receptor, two domains, long cytoplasmic tail, 3	**-6.4**
NM_019111	HLA-DRA	major histocompatibility complex, class II, DR alpha	**-6.3**
NR_047529	SIGLEC17P	sialic acid binding Ig-like lectin 17, pseudogene	**-6.2**
NM_003650	CST7	cystatin F (leukocystatin)	**-6.0**
OTTHUMT00000338835	TRGJP1	T cell receptor gamma joining P1	**-6.0**
NM_001195683	TGFBR3	transforming growth factor beta receptor III	**-6.0**

NM_030806	C1orf21	chromosome 1 open reading frame 21	**-5.8**
NM_000953	PTGDR	prostaglandin D2 receptor (DP)	**-5.7**
NM_014512	KIR2DS1	killer cell immunoglobulin-like receptor, two domains, short cytoplasmic tail, 1	**-5.6**
NM_000887	ITGAX	integrin alpha X	**-5.6**
OTTHUMT00000338399	TRGV8	T cell receptor gamma variable 8	**-5.6**
NM_015192	PLCB1	phospholipase C, beta 1 (phosphoinositide-specific)	**-5.5**
NM_001004310	FCRL6	Fc receptor-like 6	**-5.4**
NM_001256841	CD300A	CD300a molecule	**-5.4**
NM_024329	EFHD2	EF-hand domain family member D2	**-5.3**
NM_001080416	MYBL1	v-myb avian myeloblastosis viral oncogene homolog-like 1	**-5.2**
NM_152309	PIK3AP1	phosphoinositide-3-kinase adaptor protein 1	**-5.2**
NM_001081955	RGS9	regulator of G-protein signaling 9	**-5.1**
NM_012335	MYO1F	myosin IF	**-5.1**
NM_001123041	CCR2	chemokine (C-C motif) receptor 2	**-5.0**
NM_001244134	MAP3K8	mitogen-activated protein kinase kinase kinase 8	**-5.0**
NM_001081573	GAB3	GRB2-associated binding protein 3	**-5.0**
NM_001105244	PTPRM	protein tyrosine phosphatase, receptor type, M	**-4.9**
NM_152501	PYHIN1	pyrin and HIN domain family, member 1	**-4.8**
XM_011519310	MAP3K8	mitogen-activated protein kinase kinase kinase 8	**-4.8**
NM_005810	KLRG1	killer cell lectin-like receptor subfamily G, member 1	**-4.8**
NM_001171653	ZEB2	zinc finger E-box binding homeobox 2	**-4.8**
NM_001173514	TYROBP	TYRO protein tyrosine kinase binding protein	**-4.7**
NM_000634	CXCR1	chemokine (C-X-C motif) receptor 1	**-4.7**
NM_004163	RAB27B	RAB27B, member RAS oncogene family	**-4.7**
NM_001270934	JAKMIP2	janus kinase and microtubule interacting protein 2	**-4.7**
NM_001111097	LYN	LYN proto-oncogene, Src family tyrosine kinase	**-4.7**

NR_026971	A2M-AS1	A2M antisense RNA 1 (head to head)	**-4.6**
OTTHUMT00000338419	TRGV2	T cell receptor gamma variable 2	**-4.6**
OTTHUMT00000351239	TRBV6-4	T cell receptor beta variable 6-4	**-4.5**
M13231	TRGC2	T cell receptor gamma constant 2	**-4.5**
BC072387	TRGV9	T cell receptor gamma variable 9	**-4.5**
NM_001303103	TLE1	transducin-like enhancer of split 1 (E(sp1) homolog, Drosophila)	**-4.4**
NM_001291468	CCL4L2	chemokine (C-C motif) ligand 4-like 2	**-4.4**
NM_001304376	ADGRG5	adhesion G protein-coupled receptor G5	**-4.4**
NM_001301029	USP28	ubiquitin specific peptidase 28	**-4.3**
NM_152353	CLDND2	claudin domain containing 2	**-4.3**
NM_002661	PLCG2	phospholipase C, gamma 2 (phosphatidylinositol-specific)	**-4.3**
NM_001166663	CD244	CD244 molecule, natural killer cell receptor 2B4	**-4.2**
NM_004775	B4GALT6	UDP-Gal:betaGlcNAc beta 1,4-galactosyltransferase, polypeptide 6	**-4.2**
NM_003670	BHLHE40	basic helix-loop-helix family, member e40	**-4.2**
NM_001081455	P2RY14	purinergic receptor P2Y, G-protein coupled, 14	**-4.2**
NM_021822	APOBEC3G	apolipoprotein B mRNA editing enzyme, catalytic polypeptide-like 3G	**-4.2**
NM_000615	NCAM1	neural cell adhesion molecule 1	**-4.1**
OTTHUMT00000338828	TRGJP2	T cell receptor gamma joining P2	**-4.1**
NM_016616	NME8	NME/NM23 family member 8	**-4.1**
NM_014219	KIR2DL2	killer cell immunoglobulin-like receptor, two domains, long cytoplasmic tail, 2	**-4.1**
NM_001160167	PRR5L	proline rich 5 like	**-4.1**
NM_000639	FASLG	Fas ligand (TNF superfamily, member 6)	**-4.0**
NM_001288973	ADAM12	ADAM metallopeptidase domain 12	**-4.0**
OTTHUMT00000410934	TRDJ2	T cell receptor delta joining 2	**-4.0**

NM_002609	PDGFRB	platelet-derived growth factor receptor, beta polypeptide	**-4.0**
OTTHUMT00000487452	CCL4L1	chemokine (C-C motif) ligand 4-like 1	**-4.0**
NM_003373	VCL	vinculin	**-4.0**
NM_001004470	ST8SIA6	ST8 alpha-N-acetyl-neuraminide alpha-2,8-sialyltransferase 6	**-3.9**
NM_004924	ACTN4	actinin, alpha 4	**-3.9**
NM_001098484	SLC4A4	solute carrier family 4 (sodium bicarbonate cotransporter), member 4	**-3.9**
NM_181791	GPR141	G protein-coupled receptor 141	**-3.9**
NM_001278182	EOMES	eomesodermin	**-3.9**
NM_020988	GNAO1	guanine nucleotide binding protein (G protein), alpha activating activity polypeptide O	**-3.9**
NM_014762	DHCR24	24-dehydrocholesterol reductase	**-3.8**
NM_001017403	LGR6	leucine-rich repeat containing G protein-coupled receptor 6	**-3.8**
NM_006056	NMUR1	neuromedin U receptor 1	**-3.8**
NM_000619	IFNG	interferon, gamma	**-3.8**
NM_033071	SYNE1	spectrin repeat containing, nuclear envelope 1	**-3.8**
NM_001042729	FGR	FGR proto-oncogene, Src family tyrosine kinase	**-3.8**
NM_014729	TOX	thymocyte selection-associated high mobility group box	**-3.8**
NM_006682	FGL2	fibrinogen-like 2	**-3.7**
NM_000302	PLOD1	procollagen-lysine, 2-oxoglutarate 5-dioxygenase 1	**-3.7**
NM_001171171	CX3CR1	chemokine (C-X3-C motif) receptor 1	**-3.7**
NM_007053	CD160	CD160 molecule	**-3.7**
NM_001270526	STOM	stomatin	**-3.7**
NM_032428	FRMPD3	FERM and PDZ domain containing 3	**-3.7**
NM_001001523	RORC	RAR-related orphan receptor C	**-3.7**
NM_024310	PLEKHF1	pleckstrin homology domain containing, family F (with FYVE domain) member 1	**-3.7**
NM_001100398	RAP1GAP2	RAP1 GTPase activating protein 2	**-3.6**

NM_004776	B4GALT5	UDP-Gal:betaGlcNAc beta 1,4-galactosyltransferase, polypeptide 5	**-3.6**
NM_002261	KLRC3	killer cell lectin-like receptor subfamily C, member 3	**-3.6**
NM_001308315	TSPAN2	tetraspanin 2	**-3.6**
BC072387	TRGV9	T cell receptor gamma variable 9	**-3.6**
NM_001098802	CEP78	centrosomal protein 78kDa	**-3.6**
NM_001145199	C12orf75	chromosome 12 open reading frame 75	**-3.6**
NM_001145457	NCR1	natural cytotoxicity triggering receptor 1	**-3.5**
NM_001004431	METRNL	meteorin, glial cell differentiation regulator-like	**-3.5**
NM_001135685	LTK	leukocyte receptor tyrosine kinase	**-3.5**
NM_052966	FAM129A	family with sequence similarity 129, member A	**-3.5**
NM_013254	TBK1	TANK-binding kinase 1	**-3.4**
NM_001144822	CD58	CD58 molecule	**-3.4**
NM_001145466	NCR3	natural cytotoxicity triggering receptor 3	**-3.4**
NM_001311313	F2R	coagulation factor II (thrombin) receptor	**-3.4**
NM_000271	NPC1	Niemann-Pick disease, type C1	**-3.4**
NM_001142343	CMKLR1	chemerin chemokine-like receptor 1	**-3.3**
NR_004386	RNU105B	RNA, U105B small nucleolar	**-3.3**
NM_001037131	AGAP1	ArfGAP with GTPase domain, ankyrin repeat and PH domain 1	**-3.3**
BC039116	TRGC2	T cell receptor gamma constant 2	**-3.3**
NM_001162951	SYTL2	synaptotagmin-like 2	**-3.3**
NM_001282640	SUSD1	sushi domain containing 1	**-3.3**
NM_001001396	ATP2B4	ATPase, Ca++ transporting, plasma membrane 4	**-3.3**
NM_017983	WIPI1	WD repeat domain, phosphoinositide interacting 1	**-3.3**

NM_001159643	MCTP2	multiple C2 domains, transmembrane 2	**-3.3**
NM_001018011	ZBTB16	zinc finger and BTB domain containing 16	**-3.3**
NM_002964	S100A8	S100 calcium binding protein A8	**-3.3**
NM_052939	FCRL3	Fc receptor-like 3	**-3.3**
NM_020133	AGPAT4	1-acylglycerol-3-phosphate O-acyltransferase 4	**-3.2**
NM_018644	B3GAT1	beta-1,3-glucuronyltransferase 1	**-3.2**
NM_015166	MLC1	megalencephalic leukoencephalopathy with subcortical cysts 1	**-3.2**
BC073897	MXRA7	matrix-remodelling associated 7	**-3.2**
NM_145648	SLC15A4	solute carrier family 15 (oligopeptide transporter), member 4	**-3.2**
NR_029683	MIR142	microRNA 142	**-3.2**
NM_001242867	KIR3DL2	killer cell immunoglobulin-like receptor, three domains, long cytoplasmic tail, 2	**-3.2**
NM_001243794	CHST12	carbohydrate (chondroitin 4) sulfotransferase 12	**-3.2**
NM_001066	TNFRSF1B	tumor necrosis factor receptor superfamily, member 1B	**-3.2**
NM_001282399	IL18R1	interleukin 18 receptor 1	**-3.2**
NM_152280	SYT11	synaptotagmin XI	**-3.2**
NM_001433	ERN1	endoplasmic reticulum to nucleus signaling 1	**-3.2**
AF018171	YME1L1	YME1-like 1 ATPase	**-3.2**
NM_004747	DLG5	discs, large homolog 5 (Drosophila)	**-3.1**
NM_017629	AGO4	argonaute RISC catalytic component 4	**-3.1**
ENST00000610437	KIR3DL3	killer cell immunoglobulin-like receptor, three domains, long cytoplasmic tail, 3	**-3.1**
NM_001161342	TMEM171	transmembrane protein 171	**-3.1**
NM_016441	CRIM1	cysteine rich transmembrane BMP regulator 1 (chordin-like)	**-3.1**
ENST00000394409	PPP2R2B	protein phosphatase 2, regulatory subunit B, beta	**-3.1**

NM_001114173	CTSC	cathepsin C	**-3.1**
NM_012313	KIR2DS3	killer cell immunoglobulin-like receptor, two domains, short cytoplasmic tail, 3	**-3.1**
NM_001126128	PROK2	prokineticin 2	**-3.0**
NM_020733	HEG1	heart development protein with EGF-like domains 1	**-3.0**
NM_001282971	MTSS1	metastasis suppressor 1	**-3.0**
NM_001284194	FUT11	fucosyltransferase 11 (alpha (1,3) fucosyltransferase)	**-3.0**
NM_000246	CIITA	class II, major histocompatibility complex, transactivator	**-3.0**
NM_002305	LGALS1	lectin, galactoside-binding, soluble, 1	**-3.0**
NM_001039547	GK5	glycerol kinase 5 (putative)	**-3.0**
NM_000397	CYBB	cytochrome b-245, beta polypeptide	**-3.0**
NM_001282862	RASGEF1A	RasGEF domain family member 1A	**-3.0**
NR_024277	AGPAT4-IT1	AGPAT4 intronic transcript 1	**-3.0**
NM_017931	TTC38	tetratricopeptide repeat domain 38	**-3.0**
NM_022365	DNAJC1	DnaJ (Hsp40) homolog, subfamily C, member 1	**-3.0**
NM_003265	TLR3	toll-like receptor 3	**-3.0**
NR_003573	ANXA2P2	annexin A2 pseudogene 2	**-2.9**
NM_001256574	ENC1	ectodermal-neural cortex 1 (with BTB domain)	**-2.9**
NM_001198	PRDM1	PR domain containing 1, with ZNF domain	**-2.9**
NM_001300899	MYO6	myosin VI	**-2.9**
NM_199280	FAM179A	family with sequence similarity 179, member A	**-2.9**
NM_001301036	TMCC3	transmembrane and coiled-coil domain family 3	**-2.9**
NM_004227	CYTH3	cytohesin 3	**-2.9**
NM_001061	TBXAS1	thromboxane A synthase 1 (platelet)	**-2.9**
ENST00000598878	METTL6	methyltransferase like 6	**-2.9**
NM_001015002	LLGL2	lethal giant larvae homolog 2 (Drosophila)	**-2.9**
NM_021033	RAP2A	RAP2A, member of RAS oncogene family	**-2.9**
NM_001104544	TMEM255A	transmembrane protein 255A	**-2.9**

NM_001142650	HNRNPLL	heterogeneous nuclear ribonucleoprotein L-like	**-2.8**
NM_024630	ZDHHC14	zinc finger, DHHC-type containing 14	**-2.8**
NM_001304448	KLRC1	killer cell lectin-like receptor subfamily C, member 1	**-2.8**
XR_251175	FAR2P3	fatty acyl-CoA reductase 2 pseudogene 3	**-2.8**
NM_015175	NBEAL2	neurobeachin like 2	**-2.8**
NM_020531	APMAP	adipocyte plasma membrane associated protein	**-2.8**
NM_001290072	ENPP5	ectonucleotide pyrophosphatase/phosphodiesterase 5 (putative)	**-2.8**
NM_014508	APOBEC3C	apolipoprotein B mRNA editing enzyme, catalytic polypeptide-like 3C	**-2.8**
NM_001261825	OASL	2-5-oligoadenylate synthetase-like	**-2.7**
NR_030618	MIR873	microRNA 873	**-2.7**
NM_025208	PDGFD	platelet derived growth factor D	**-2.7**
NM_001308147	PLEKHG3	pleckstrin homology domain containing, family G (with RhoGef domain) member 3	**-2.7**
NM_001013627	NHSL2	NHS-like 2	**-2.7**
NM_001134486	GBP5	guanylate binding protein 5	**-2.7**
NM_182523	CMC1	C-x(9)-C motif containing 1	**-2.7**
NM_024940	DOCK5	dedicator of cytokinesis 5	**-2.7**
NM_024830	LPCAT1	lysophosphatidylcholine acyltransferase 1	**-2.7**
NM_001131008	PTPN12	protein tyrosine phosphatase, non-receptor type 12	**-2.7**
NR_120609	GOLGA2P6	golgin A2 pseudogene 6	**-2.7**
NM_001145112	PATL2	protein associated with topoisomerase II homolog 2 (yeast)	**-2.7**
NM_001135186	ABI3	ABI family, member 3	**-2.6**
NM_018181	ZNF532	zinc finger protein 532	**-2.6**
NM_001114759	ZNF683	zinc finger protein 683	**-2.6**
NM_001193431	PTPN22	protein tyrosine phosphatase, non-receptor type	**-2.6**

		22 (lymphoid)	
NM_001282663	MICAL2	microtubule associated monooxygenase, calponin and LIM domain containing 2	**-2.6**
NM_001033044	GLUL	glutamate-ammonia ligase	**-2.6**
NM_001258214	IL12RB2	interleukin 12 receptor, beta 2	**-2.6**
NM_001277201	SIGLEC7	sialic acid binding Ig-like lectin 7	**-2.6**
NM_002104	GZMK	granzyme K	**-2.6**
NM_003974	DOK2	docking protein 2	**-2.6**
NM_000074	CD40LG	CD40 ligand	**-2.6**
NM_003608	GPR65	G protein-coupled receptor 65	**-2.6**
NM_001127714	HIVEP3	human immunodeficiency virus type I enhancer binding protein 3	**-2.6**
NM_001143976	WEE1	WEE1 G2 checkpoint kinase	**-2.6**
NM_022140	EPB41L4A	erythrocyte membrane protein band 4.1 like 4A	**-2.6**
NM_002378	MATK	megakaryocyte-associated tyrosine kinase	**-2.6**
NM_013308	GPR171	G protein-coupled receptor 171	**-2.6**
NM_018438	FBXO6	F-box protein 6	**-2.6**
NM_001135731	ZMAT4	zinc finger, matrin-type 4	**-2.6**
NR_026716	KIR3DX1	killer cell immunoglobulin-like receptor, three domains, X1	**-2.6**
NM_005677	COLQ	collagen-like tail subunit (single strand of homotrimer) of asymmetric acetylcholinesterase	**-2.6**
NM_014572	LATS2	large tumor suppressor kinase 2	**-2.5**
NM_002260	KLRC2	killer cell lectin-like receptor subfamily C, member 2	**-2.5**
NM_001289999	NFIL3	nuclear factor, interleukin 3 regulated	**-2.5**
NM_001261835	BZRAP1	benzodiazepine receptor (peripheral) associated protein 1	**-2.5**
NM_005907	MAN1A1	mannosidase, alpha, class 1A, member 1	**-2.5**
NM_001278736	CCL5	chemokine (C-C motif) ligand 5	**-2.5**
NM_003246	THBS1	thrombospondin 1	**-2.5**
NM_173483	CYP4F22	cytochrome P450, family 4, subfamily F, polypeptide 22	**-2.5**

NM_020453	ATP10D	ATPase, class V, type 10D	**-2.5**
NM_001173463	KIF21A	kinesin family member 21A	**-2.5**
NM_004106	FCER1G	Fc fragment of IgE, high affinity I, receptor for; gamma polypeptide	**-2.5**
NM_004482	GALNT3	polypeptide N-acetylgalactosaminyltransferase 3	**-2.5**
NM_001303420	COLGALT2	collagen beta(1-O)galactosyltransferase 2	**-2.5**
NM_001098503	PTPRJ	protein tyrosine phosphatase, receptor type, J	**-2.5**
NM_001190821	SMAD7	SMAD family member 7	**-2.5**
OTTHUMT00000338832	TRGV10	T cell receptor gamma variable 10 (non-functional)	**-2.5**
NM_012289	KEAP1	kelch-like ECH-associated protein 1	**-2.5**
NM_001335	CTSW	cathepsin W	**-2.5**
NM_001114108	TTC22	tetratricopeptide repeat domain 22	**-2.4**
NM_004580	RAB27A	RAB27A, member RAS oncogene family	**-2.4**
NM_152781	HEATR9	HEAT repeat containing 9	**-2.4**
NM_001079526	IKZF2	IKAROS family zinc finger 2	**-2.4**
NM_013313	YPEL1	yippee like 1	**-2.4**
NM_001100164	PHACTR2	phosphatase and actin regulator 2	**-2.4**
NM_001146032	FCHO2	FCH domain only 2	**-2.4**
NR_028045	KLRAP1	killer cell lectin-like receptor subfamily A pseudogene 1	**-2.4**
NM_153698	AAED1	AhpC/TSA antioxidant enzyme domain containing 1	**-2.4**
NM_003037	SLAMF1	signaling lymphocytic activation molecule family member 1	**-2.4**
NM_001191	BCL2L1	BCL2-like 1	**-2.4**
NM_001079874	VAV3	vav 3 guanine nucleotide exchange factor	**-2.4**
NM_001017373	SAMD3	sterile alpha motif domain containing 3	**-2.4**
NM_001145088	TBC1D31	TBC1 domain family, member 31	**-2.3**
OTTHUMT00000401873	TRAV1-2	T cell receptor alpha variable 1-2	**-2.3**

NR_039930	MIR4772	microRNA 4772	**-2.3**
NM_001290023	IL12RB1	interleukin 12 receptor, beta 1	**-2.3**
NM_001243198	HIP1	huntingtin interacting protein 1	**-2.3**
NM_006564	CXCR6	chemokine (C-X-C motif) receptor 6	**-2.3**
NM_001136498	CISD3	CDGSH iron sulfur domain 3	**-2.3**
NM_001287010	GLIPR2	GLI pathogenesis-related 2	**-2.3**
NM_001127231	AUTS2	autism susceptibility candidate 2	**-2.3**
NM_001195059	PLEKHO2	pleckstrin homology domain containing, family O member 2	**-2.3**
NM_004669	CLIC3	chloride intracellular channel 3	**-2.3**
NM_001316676	PTPRE	protein tyrosine phosphatase, receptor type, E	**-2.3**
NM_015916	CALHM2	calcium homeostasis modulator 2	**-2.3**
NM_001197293	DPYSL2	dihydropyrimidinase-like 2	**-2.3**
NR_033651	MSC-AS1	MSC antisense RNA 1	**-2.3**
NM_024430	PSTPIP2	proline-serine-threonine phosphatase interacting protein 2	**-2.3**
NM_000416	IFNGR1	interferon gamma receptor 1	**-2.3**
NM_000239	LYZ	lysozyme	**-2.3**
NM_016463	CXXC5	CXXC finger protein 5	**-2.3**
NM_006111	ACAA2	acetyl-CoA acyltransferase 2	**-2.3**
NM_001166002	APOBEC3H	apolipoprotein B mRNA editing enzyme, catalytic polypeptide-like 3H	**-2.3**
NM_001223	CASP1	caspase 1	**-2.3**
NM_001184720	GYG1	glycogenin 1	**-2.3**
NR_073415	HSP90AB4P	heat shock protein 90kDa alpha (cytosolic), class B member 4, pseudogene	**-2.3**
NM_001122898	CD99	CD99 molecule	**-2.3**
NM_000387	SLC25A20	solute carrier family 25 (carnitine/acylcarnitine translocase), member 20	**-2.2**
NM_001116	ADCY9	adenylate cyclase 9	**-2.2**
NM_001099433	JAKMIP1	janus kinase and microtubule interacting protein 1	**-2.2**

NM_000153	GALC	galactosylceramidase	**-2.2**
NM_001130831	GAS7	growth arrest-specific 7	**-2.2**
NM_001135553	MKNK1	MAP kinase interacting serine/threonine kinase 1	**-2.2**
NM_001161748	LIM2	lens intrinsic membrane protein 2	**-2.2**
NM_001005498	RHBDF2	rhomboid 5 homolog 2 (Drosophila)	**-2.2**
NM_001282736	PLXDC2	plexin domain containing 2	**-2.2**
NM_003807	TNFSF14	tumor necrosis factor (ligand) superfamily, member 14	**-2.2**
NM_024605	ARHGAP10	Rho GTPase activating protein 10	**-2.2**
NM_001006636	GTDC1	glycosyltransferase like domain containing 1	**-2.2**
NM_198485	TPRG1	tumor protein p63 regulated 1	**-2.2**
NM_001257389	CD63	CD63 molecule	**-2.2**
NM_020856	TSHZ3	teashirt zinc finger homeobox 3	**-2.2**
NM_004419	DUSP5	dual specificity phosphatase 5	**-2.2**
NM_000954	PTGDS	prostaglandin D2 synthase 21kDa (brain)	**-2.2**
NM_002432	MNDA	myeloid cell nuclear differentiation antigen	**-2.2**
NM_001293178	PRSS23	protease, serine, 23	**-2.2**
NR_006881	SNORD3C	small nucleolar RNA, C/D box 3C	**-2.2**
NM_003175	XCL2	chemokine (C motif) ligand 2	**-2.2**
NM_001010863	C10orf128	chromosome 10 open reading frame 128	**-2.2**
NM_001177506	AOAH	acyloxyacyl hydrolase (neutrophil)	**-2.2**
NM_001142339	GNAL	guanine nucleotide binding protein (G protein), alpha activating activity polypeptide, olfactory type	**-2.2**
NM_001154	ANXA5	annexin A5	**-2.2**
NM_001244871	DAB2	Dab, mitogen-responsive phosphoprotein, homolog 2 (Drosophila)	**-2.2**
NM_014661	FAM53B	family with sequence similarity 53, member B	**-2.2**
XM_011530934	NHSL2	NHS-like 2	**-2.2**

NM_004054	C3AR1	complement component 3a receptor 1	**-2.2**
NM_001198665	ARHGEF12	Rho guanine nucleotide exchange factor (GEF) 12	**-2.2**
NM_004522	KIF5C	kinesin family member 5C	**-2.2**
NM_001111307	PDE4A	phosphodiesterase 4A, cAMP-specific	**-2.2**
NM_001303126	RHEBL1	Ras homolog enriched in brain like 1	**-2.1**
NM_138801	GALM	galactose mutarotase (aldose 1-epimerase)	**-2.1**
NM_001669	ARSD	arylsulfatase D	**-2.1**
NM_198147	ABHD15	abhydrolase domain containing 15	**-2.1**
NM_001145725	LYAR	Ly1 antibody reactive	**-2.1**
NM_001291695	KIR2DS2	killer cell immunoglobulin-like receptor, two domains, short cytoplasmic tail, 2	**-2.1**
NM_033515	ARHGAP18	Rho GTPase activating protein 18	**-2.1**
NM_006332	IFI30	interferon, gamma-inducible protein 30	**-2.1**
NM_001083539	KIR3DS1	killer cell immunoglobulin-like receptor, three domains, short cytoplasmic tail, 1	**-2.1**
NM_001110556	FLNA	filamin A, alpha	**-2.1**
NR_003341	SNORD116-27	small nucleolar RNA, C/D box 116-27	**-2.1**
NM_003764	STX11	syntaxin 11	**-2.1**
NR_040102	FLJ33581	uncharacterized LOC400839	**-2.1**
NM_014957	DENND3	DENN/MADD domain containing 3	**-2.1**
NM_002395	ME1	malic enzyme 1, NADP(+)-dependent, cytosolic	**-2.1**
NM_015230	ARAP2	ArfGAP with RhoGAP domain, ankyrin repeat and PH domain 2	**-2.1**

NM_001775	CD38	CD38 molecule	**-2.1**
NM_001114380	ITGAL	integrin alpha L	**-2.1**
NM_001184714	SLAMF6	SLAM family member 6	**-2.1**
NM_001136190	LAX1	lymphocyte transmembrane adaptor 1	**-2.1**
NM_006750	SNTB2	syntrophin, beta 2 (dystrophin-associated protein A1, 59kDa, basic component 2)	**-2.1**
NM_000564	IL5RA	interleukin 5 receptor, alpha	**-2.1**
NM_031459	SESN2	sestrin 2	**-2.1**
OTTHUMT00000401536	TRDV3	T cell receptor delta variable 3	**-2.1**
NM_001195328	RAB9A	RAB9A, member RAS oncogene family	**-2.1**
NM_014822	SEC24D	SEC24 homolog D, COPII coat complex component	**-2.1**
NR_036188	MIR4302	microRNA 4302	**-2.1**
NR_046764	AOAH-IT1	AOAH intronic transcript 1	**-2.1**
NM_006871	RIPK3	receptor-interacting serine-threonine kinase 3	**-2.1**
NM_001204897	TM4SF19	transmembrane 4 L six family member 19	**-2.1**
NM_001114172	PIK3R3	phosphoinositide-3-kinase, regulatory subunit 3 (gamma)	**-2.1**
NM_014899	RHOBTB3	Rho-related BTB domain containing 3	**-2.1**
NM_014218	KIR2DL1	killer cell immunoglobulin-like receptor, two domains, long cytoplasmic tail, 1	**-2.1**
NM_001109	ADAM8	ADAM metallopeptidase domain 8	**-2.1**
NM_133494	NEK7	NIMA-related kinase 7	**-2.1**
NM_001193380	IL17RE	interleukin 17 receptor E	**-2.0**

NM_001620	AHNAK	AHNAK nucleoprotein	**-2.0**
NM_001128325	SPON2	spondin 2, extracellular matrix protein	**-2.0**
XM_011521475	ANXA2	annexin A2	**-2.0**
NM_001040697	UEVLD	UEV and lactate/malate dehyrogenase domains	**-2.0**
NM_001242628	GFOD1	glucose-fructose oxidoreductase domain containing 1	**-2.0**
NM_001008949	ITPRIPL1	inositol 1,4,5-trisphosphate receptor interacting protein-like 1	**-2.0**
NM_001142523	IRAK3	interleukin 1 receptor associated kinase 3	**-2.0**
NM_001130110	SETBP1	SET binding protein 1	**-2.0**
NM_001005862	ERBB2	erb-b2 receptor tyrosine kinase 2	**-2.0**
NM_003870	IQGAP1	IQ motif containing GTPase activating protein 1	**-2.0**
NM_013233	STK39	serine threonine kinase 39	**-2.0**
NM_021156	TMX4	thioredoxin-related transmembrane protein 4	**-2.0**
NM_001099455	CPPED1	calcineurin-like phosphoesterase domain containing 1	**-2.0**
NM_001195243	SMKR1	small lysine-rich protein 1	**-2.0**
NM_015180	SYNE2	spectrin repeat containing, nuclear envelope 2	**-2.0**
NM_001253835	IGFBP7	insulin like growth factor binding protein 7	**-2.0**
NM_175617	MT1E	metallothionein 1E	**-2.0**
NM_001143821	PLEKHA5	pleckstrin homology domain containing, family A member 5	**-2.0**
NM_000594	TNF	tumor necrosis factor	**-2.0**
NM_001303512	PDZD4	PDZ domain containing 4	**-2.0**

NM_024576	OGFRL1	opioid growth factor receptor-like 1	**-2.0**
NM_012383	OSTF1	osteoclast stimulating factor 1	**-2.0**
NM_001278557	WSB2	WD repeat and SOCS box containing 2	**-2.0**
NM_000878	IL2RB	interleukin 2 receptor, beta	**-2.0**
NM_001198801	EIF4G3	eukaryotic translation initiation factor 4 gamma, 3	**-2.0**
NM_001039477	THEMIS2	thymocyte selection associated family member 2	**-2.0**
NM_001123375	HIST2H3D	histone cluster 2, H3d	**-2.0**
NM_006786	UTS2	urotensin 2	**-2.0**
NM_000101	CYBA	cytochrome b-245, alpha polypeptide	**-2.0**
NM_002121	HLA-DPB1	major histocompatibility complex, class II, DP beta 1	**-2.0**
NM_001042473	ACBD5	acyl-CoA binding domain containing 5	**-2.0**
NR_108036	CFAP58-AS1	CFAP58 antisense RNA 1 (head to head)	**-2.0**
NM_001206567	IFI16	interferon, gamma-inducible protein 16	**-2.0**
NM_001008211	OPTN	optineurin	**-2.0**
NM_015550	OSBPL3	oxysterol binding protein-like 3	**-2.0**
NM_001042576	RRBP1	ribosome binding protein 1	**-2.0**
NM_001013742	DGKK	diacylglycerol kinase, kappa	**-2.0**
NM_001206957	RASSF1	Ras association (RalGDS/AF-6) domain family member 1	**-2.0**
NM_001204406	ALOX5AP	arachidonate 5-lipoxygenase-activating protein	**-2.0**
NM_145798	OSBPL7	oxysterol binding protein-like 7	**-2.0**

NM_001100422	SPATS2L	spermatogenesis associated, serine-rich 2-like	**-2.0**
NM_001009998	SSBP4	single stranded DNA binding protein 4	**-2.0**
NM_001285460	IQGAP2	IQ motif containing GTPase activating protein 2	**-2.0**
NM_001171971	CDHR1	cadherin-related family member 1	**-2.0**
NM_001199775	CPD	carboxypeptidase D	**-2.0**
NM_021205	RHOU	ras homolog family member U	**-2.0**
NM_001142276	APLP2	amyloid beta (A4) precursor-like protein 2	**-2.0**
NM_001084	PLOD3	procollagen-lysine, 2-oxoglutarate 5-dioxygenase 3	**-2.0**
NM_001172411	VANGL1	VANGL planar cell polarity protein 1	**-2.0**
NM_000527	LDLR	low density lipoprotein receptor	**-2.0**
NM_001030288	SPN	sialophorin	**-1.9**
NM_001130916	TGFBR1	transforming growth factor, beta receptor 1	**-1.9**
NM_001606	ABCA2	ATP binding cassette subfamily A member 2	**-1.9**
NM_006851	GLIPR1	GLI pathogenesis-related 1	**-1.9**
NM_004346	CASP3	caspase 3	**-1.9**
NM_001297576	PEA15	phosphoprotein enriched in astrocytes 15	**-1.9**
NM_013368	SERTAD3	SERTA domain containing 3	**-1.9**
NM_001142864	PIEZO1	piezo-type mechanosensitive ion channel component 1	**-1.9**
NM_001128203	PLA2G16	phospholipase A2, group XVI	**-1.9**
OTTHUMT00000060170	CHST12	carbohydrate (chondroitin 4) sulfotransferase 12	**-1.9**

NM_001172623	NEO1	neogenin 1	**-1.9**
NM_001283018	NAPB	N-ethylmaleimide-sensitive factor attachment protein, beta	**-1.9**
NM_001178138	TFDP2	transcription factor Dp-2 (E2F dimerization partner 2)	**-1.9**
NM_198457	ZNF600	zinc finger protein 600	**-1.9**
NM_000944	PPP3CA	protein phosphatase 3, catalytic subunit, alpha isozyme	**-1.9**
NM_001145144	SLC1A5	solute carrier family 1 (neutral amino acid transporter), member 5	**-1.9**
NM_001145044	SLCO3A1	solute carrier organic anion transporter family, member 3A1	**-1.9**
NM_152426	APOBEC3D	apolipoprotein B mRNA editing enzyme, catalytic polypeptide-like 3D	**-1.9**
NM_002124	HLA-DRB1	major histocompatibility complex, class II, DR beta 1	**-1.9**
NM_030978	ARPC5L	actin related protein 2/3 complex subunit 5-like	**-1.9**
NM_017439	GSAP	gamma-secretase activating protein	**-1.9**
NM_005103	FEZ1	fasciculation and elongation protein zeta 1	**-1.9**
NM_001102592	HENMT1	HEN1 methyltransferase homolog 1 (Arabidopsis)	**-1.9**
NM_001244938	TXN	thioredoxin	**-1.9**
NM_153690	FAM43A	family with sequence similarity 43, member A	**-1.9**
NM_007207	DUSP10	dual specificity phosphatase 10	**-1.9**
NM_006399	BATF	basic leucine zipper transcription factor, ATF-like	**-1.9**
NM_001199797	PTPN7	protein tyrosine phosphatase, non-receptor type 7	**-1.9**
NM_001007269	GEMIN7	gem nuclear organelle associated protein 7	**-1.9**
NM_005313	PDIA3	protein disulfide isomerase family A member 3	**-1.9**

NM_001135052	SYK	spleen tyrosine kinase	**-1.9**
NM_001003962	CAPNS1	calpain, small subunit 1	**-1.9**
NM_001311	CRIP1	cysteine-rich protein 1 (intestinal)	**-1.9**
NM_001122681	SH3BP2	SH3-domain binding protein 2	**-1.9**
NM_018990	SASH3	SAM and SH3 domain containing 3	**-1.9**
NM_024837	ATP8B4	ATPase, class I, type 8B, member 4	**-1.9**
NM_138441	MB21D1	Mab-21 domain containing 1	**-1.9**
NM_001286715	AGTPBP1	ATP/GTP binding protein 1	**-1.9**
NM_001042368	RALGDS	ral guanine nucleotide dissociation stimulator	**-1.9**
NM_001160417	ZBP1	Z-DNA binding protein 1	**-1.9**
NM_005415	SLC20A1	solute carrier family 20 (phosphate transporter), member 1	**-1.9**
NM_001080480	MBOAT1	membrane bound O-acyltransferase domain containing 1	**-1.9**
NM_020823	TMEM181	transmembrane protein 181	**-1.9**
NM_018998	FBXW5	F-box and WD repeat domain containing 5	**-1.9**
NM_000876	IGF2R	insulin-like growth factor 2 receptor	**-1.9**
NM_018690	APOBR	apolipoprotein B receptor	**-1.9**
NM_001001437	CCL3L3	chemokine (C-C motif) ligand 3-like 3	**-1.8**
NM_001042685	LGALS9B	lectin, galactoside-binding, soluble, 9B	**-1.8**
NM_001174166	SLC16A6	solute carrier family 16, member 6	**-1.8**
NM_000637	GSR	glutathione reductase	**-1.8**

NM_001130721	ELOVL6	ELOVL fatty acid elongase 6	**-1.8**
NM_000576	IL1B	interleukin 1 beta	**-1.8**
NM_001289823	FURIN	furin (paired basic amino acid cleaving enzyme)	**-1.8**
NM_032016	STARD3NL	STARD3 N-terminal like	**-1.8**
NM_000660	TGFB1	transforming growth factor beta 1	**-1.8**
NM_001161441	SH2D2A	SH2 domain containing 2A	**-1.8**
NM_014326	DAPK2	death-associated protein kinase 2	**-1.8**
NM_003521	HIST1H2BM	histone cluster 1, H2bm	**-1.8**
NM_000332	ATXN1	ataxin 1	**-1.8**
NM_001304275	RAPGEF1	Rap guanine nucleotide exchange factor 1	**-1.8**
NM_001127183	CFLAR	CASP8 and FADD like apoptosis regulator	**-1.8**
NM_001127197	ELF4	E74-like factor 4 (ets domain transcription factor)	**-1.8**
NM_002727	SRGN	serglycin	**-1.8**
NM_000259	MYO5A	myosin VA	**-1.8**
NM_001035260	VPS26A	VPS26 retromer complex component A	**-1.8**
NM_004759	MAPKAPK2	mitogen-activated protein kinase-activated protein kinase 2	**-1.8**
NM_002211	ITGB1	integrin beta 1	**-1.8**
NM_138426	GLCCI1	glucocorticoid induced 1	**-1.8**
NM_001206651	SH3GLB1	SH3-domain GRB2-like endophilin B1	**-1.8**
NM_001308445	NAA50	N(alpha)-acetyltransferase 50, NatE catalytic subunit	**-1.8**

NM_000585	IL15	interleukin 15	**-1.8**
NM_004252	SLC9A3R1	solute carrier family 9, subfamily A (NHE3, cation proton antiporter 3), member 3 regulator 1	**-1.8**
NM_000956	PTGER2	prostaglandin E receptor 2	**-1.8**
NM_005923	MAP3K5	mitogen-activated protein kinase kinase kinase 5	**-1.8**
NM_001005176	SP140	SP140 nuclear body protein	**-1.8**
NM_033054	MYO1G	myosin IG	**-1.8**
NM_001127361	RNF19B	ring finger protein 19B	**-1.8**
NM_001030272	ARNTL	aryl hydrocarbon receptor nuclear translocator-like	**-1.8**
NM_001204426	LIMK1	LIM domain kinase 1	**-1.8**
NM_001009991	SYTL3	synaptotagmin-like 3	**-1.8**
NR_038993	ZBTB20-AS1	ZBTB20 antisense RNA 1	**-1.8**
NM_005345	HSPA1A	heat shock 70kDa protein 1A	**-1.8**
NM_013436	NCKAP1	NCK-associated protein 1	**-1.8**
NM_001033553	SPECC1	sperm antigen with calponin homology and coiled-coil domains 1	**-1.8**
NM_001042440	CAST	calpastatin	**-1.8**
NM_001256763	FAM49B	family with sequence similarity 49, member B	**-1.8**
NM_005127	CLEC2B	C-type lectin domain family 2, member B	**-1.8**
NM_001135095	FNDC3B	fibronectin type III domain containing 3B	**-1.8**
NM_000919	PAM	peptidylglycine alpha-amidating monooxygenase	**-1.8**
NM_001690	ATP6V1A	ATPase, H+ transporting, lysosomal 70kDa, V1 subunit A	**-1.8**

NM_018268	WDR41	WD repeat domain 41	**-1.8**
NM_001282710	PQLC3	PQ loop repeat containing 3	**-1.8**
NM_006826	YWHAQ	tyrosine 3-monooxygenase/tryptophan 5-monooxygenase activation protein, theta	**-1.8**
ENST00000564037	GBP3	guanylate binding protein 3	**-1.8**
NM_001032282	KLF10	Kruppel-like factor 10	**-1.7**
NM_016641	GDE1	glycerophosphodiester phosphodiesterase 1	**-1.7**
NM_198321	GALNT10	polypeptide N-acetylgalactosaminyltransferase 10	**-1.7**
NM_001195215	DENND1B	DENN/MADD domain containing 1B	**-1.7**
NM_001195200	CCDC107	coiled-coil domain containing 107	**-1.7**
NM_001301098	ZBTB44	zinc finger and BTB domain containing 44	**-1.7**
NM_016626	MEX3C	mex-3 RNA binding family member C	**-1.7**
NM_001136021	NFATC2	nuclear factor of activated T-cells, cytoplasmic, calcineurin-dependent 2	**-1.7**
NM_006729	DIAPH2	diaphanous-related formin 2	**-1.7**
NR_026790	HCG11	HLA complex group 11 (non-protein coding)	**-1.7**
NM_000700	ANXA1	annexin A1	**-1.7**
NM_002907	RECQL	RecQ helicase-like	**-1.7**
NM_001134870	PPP1R18	protein phosphatase 1, regulatory subunit 18	**-1.7**
NM_001146029	SEMA7A	semaphorin 7A, GPI membrane anchor (John Milton Hagen blood group)	**-1.7**
NM_001008660	PICALM	phosphatidylinositol binding clathrin assembly protein	**-1.7**
NM_001303618	CD226	CD226 molecule	**-1.7**

NM_002444	MSN	moesin	**-1.7**
NR_003271	SNORD3B-1	small nucleolar RNA, C/D box 3B-1	**-1.7**
NR_003271	SNORD3B-1	small nucleolar RNA, C/D box 3B-1	**-1.7**
NM_001284308	ADAP1	ArfGAP with dual PH domains 1	**-1.7**
NR_006880	SNORD3A	small nucleolar RNA, C/D box 3A	**-1.7**
NR_045116	C5orf56	chromosome 5 open reading frame 56	**-1.7**
NM_003884	KAT2B	K(lysine) acetyltransferase 2B	**-1.7**
NM_198281	GPRIN3	GPRIN family member 3	**-1.7**
NR_026755	CYP4F29P	cytochrome P450, family 4, subfamily F, polypeptide 29, pseudogene	**-1.7**
NM_015016	MAST3	microtubule associated serine/threonine kinase 3	**-1.7**
NM_001178055	PARP8	poly(ADP-ribose) polymerase family member 8	**-1.7**
NR_030374	MIR644A	microRNA 644a	**-1.7**
NM_000043	FAS	Fas cell surface death receptor	**-1.7**
NM_002003	FCN1	ficolin (collagen/fibrinogen domain containing) 1	**-1.7**
NM_001280539	RNF19A	ring finger protein 19A, RBR E3 ubiquitin protein ligase	**-1.6**
NM_001257137	ITCH	itchy E3 ubiquitin protein ligase	**-1.6**
NM_003901	SGPL1	sphingosine-1-phosphate lyase 1	**-1.6**
NM_020857	VPS18	VPS18 CORVET/HOPS core subunit	**-1.6**
NM_001270679	MCU	mitochondrial calcium uniporter	**-1.6**
NM_194291	TMEM65	transmembrane protein 65	**-1.6**

NM_153259	MCOLN2	mucolipin 2	**-1.5**
NM_001284338	NEDD4	neural precursor cell expressed, developmentally down-regulated 4, E3 ubiquitin protein ligase	**-1.5**
NM_003486	SLC7A5	solute carrier family 7 (amino acid transporter light chain, L system), member 5	**-1.5**
NM_001312653	HIST1H2BK	histone cluster 1, H2bk	**-1.5**
